# 2024 APA Annual Meeting

**DOI:** 10.1176/rcp2.70006

**Published:** 2025-03-31

The following posters were presented at the 2024 Annual Meeting of the American Psychiatric Association.

## Advancing Measurement‐Based Care: Implementation of an Open‐Source Electronic Patient‐Reported Outcomes System Across Outpatient Psychiatric Settings

Emily Berich‐Anastasio, MPH LGPC^1^, Amber Bailey, MHS^1^, Alexander Maclay, BS^1^, Robert J. Schloesser, MD^1,2^

^1^Sheppard Pratt, Baltimore, Maryland;

^2^University of Maryland, Department of Psychiatry, Baltimore, Maryland

**Background:** Measurement‐based care (MBC), particularly use of patient‐reported outcomes (PRO), has demonstrated effectiveness in mental health care with symptom and treatment monitoring. Despite evidence of their efficacy and endorsement from major organizations, obstacles to implementing PROs in mental health care persist. Certain barriers can be mitigated with the adoption of electronic PROs (ePROs). Various implementation science frameworks may aid in establishing ePROs at the point‐of‐care. These frameworks allow for structured implementation procedures, consistent evaluation, collection of qualitative and quantitative data, and opportunities to adapt or modify ePRO systems.

**Methods:** The Sheppard Pratt Health System (SPHS) has developed a standardized ePRO system to implement measurement‐based care; Point‐of‐Care Dashboards, Analysis, and Reporting Toolkit (PoCDART). This open‐source system aims to support routine collection and analysis of ePROs, expand the current ePRO system, optimize staff training and workflows to support ePRO usage, and enhance the patient experience of ePROs with brief, time efficient measures and a user‐friendly interface. Implementation of ePROs took place at multiple outpatient clinics with distinct clinical populations and operational profiles. Balasubramanian’s Learning Evaluation (LE) framework and Glasgow’s RE‐AIM were used to guide the implementation and evaluation of ePRO usage. Contextual and explanatory factors related to ePRO implementation and effects on patient outcomes were captured. Regular learning meetings and a support ticketing system allowed for continuous audit and feedback from stakeholders. User feedback was systematically captured using field notes and a support tracking database; modifications were developed and prioritized through a governance process. Small, rapid Plan‐Do‐Study‐Act (PDSA) cycles at each study site facilitated ideas for modifications. Modifications were documented using FRAME and developed using agile methodology. Implementation and outcome metrics based on RE‐AIM evaluated the process over time.

**Results:** Over 2023, over 14 thousand ePRO batteries have been completed in these outpatient clinics. 51% (n=6095) patients have completed at least one ePRO, while 25% (n=1548) have completed longitudinal ePROs. 8852 instances of ePRO documentation in the EMR by a clinician have occurred. Exploratory data analyses have elucidated differences of the characteristics of patients’ ePRO completion based on various demographic data (e.g., age, gender, race, etc.).

**Conclusion:** Using the principles of LE, RE‐AIM, and PDSA, deployment of an automated measurement based care system in outpatient psychiatric clinical settings has been successful. Ongoing use of this system at the point‐of‐care demonstrates its sustained effectiveness.

**Keywords:** Psychometrics; Measurement Based Care; Patient‐Reported Outcome Measures

## Anxiolytic Consumption During Periods of Stress: Do Medical Students Self‐Medicate?

Amalesh Honnekeri^1^, Nachiket Bhaveshaikh^2^, Usha Nayak^3^

^1^MBBS Degree Program, K.J. Somaiya Medical College, Mumbai

^2^Department of Pharmacology, K.J. Somaiya Medical College, Mumbai

^3^Department of Pharmacology, K.J. Somaiya Medical College, Mumbai

**Background:** While numerous studies have established a high prevalence of psychological stress among medical students globally, there remains a lack of data regarding their patterns of self‐medicating with anxiolytics. This study explored various trends pertaining to this practice among medical students in Mumbai.

**Methods:** Institutional Ethics Committee approval was obtained prior to the commencement of this study. Online material was administered to 180 medical students enrolled in different years of medical education in Mumbai, India. This material included an information sheet, an informed consent form, a pre‐validated questionnaire, and the Perceived Stress Scale (PSS 10). Anonymity and confidentiality were assured to the participants. As per the PSS 10, scores ranging from 0 to 13 indicated low stress, from 14 to 26 indicated moderate stress, and from 27 to 40 indicated high stress. The other questionnaire contained objective questions. Data were imported into Microsoft Excel sheets from Google forms and analyzed using the Statistical Package for Social Sciences ‐ Version 15.0.

**Results:** Analysis of the PSS 10 administered to this cohort revealed that 16% of the study subjects had experienced high perceived stress, 61% had experienced moderate perceived stress, and 23% had experienced low perceived stress. Further evaluation via the objective questionnaire showed us that 14% study respondents had considered using an anxiolytic without being prescribed one, and 6% respondents had made actual attempts to obtain anxiolytics without licensed medical supervision. 49% study subjects felt that they had neglected their health and wellness due to psychological stress. Examinations, academic burden, and apprehensions about the future were noted to be the most common causes of stress in the cohort. In the previous month, it was found that 2% study subjects had self‐medicated with anxiolytics thrice a week or more, 2% had self‐medicated with anxiolytics between 1‐2 times a week, 1% had self‐medicated with anxiolytics once in two weeks, and 5% had self‐medicated with anxiolytics once in the entire month.

**Conclusion:** This study revealed an alarming trend of consumption of anxiolytics by medical students in the absence of appropriate medical advice and supervision. It not only established the prevalence of psychological stress in the cohort, but also delved further into the circumstances surrounding which medical students experience stress, as well as the intricate patterns of self‐medicating with anxiolytics during stressful periods. This study thus points towards a worrisome reality and the need to have stronger monitoring systems in place to check this practice, while adding a new dimension to existing literature. This was a sub study of a larger study that assessed a broader spectrum of self‐medication trends and practices among medical students in Mumbai which received the Short‐Term Studentship (STS) from the Indian Council of Medical Research (ICMR).

**Keywords:** anxiolytics; medical students; stress; medical education

## Efficacy of Sulbutiamine in Patients With Attention‐Deficit Hyperactivity Disorder Clinical Observations

Hemlata Charitar, M.D., PhD

WELLKIN hospital Moka, Mauritius

**Introduction:** Sulbutiamine is a thiamine derivative developed in Japan in the mid‐60's as a Beri‐beri treatment drug (1). Since then several potential advantages have been described. Thiamine is essential for the brain and is involved in many cellular processes (energy metabolism, cell survival, antioxidant activity).

**Objective:** The goal of the clinical observations was to establish the efficacy of sulbutiamine in patients with ADHD in a private outpatient setting as stimulants were not available for a certain period of time.

**Methods:** A total of 15 adult patients who have been previously diagnosed with ADHD were observed during a 12 week period. Treatment was initiated with sulbutiamine tablets 200 mg twice per day. Clinical global impression scale was used to assess outcome of treatment. Both male and female patients were included and were aged between 18 to 43 years old. They were all previously diagnosed with Attention‐deficit hyperactivity disorder according to the DSM‐V criteria. Patients with psychiatric comorbidities were also included.

**Results:** The majority of the patients were males (60%), 40% of the patients were females. The mean age of the patients was 28.3 years. 40% of the patients were diagnosed with ADHD combined type, 60% of the patients were diagnosed with ADHD, Inattentive type. There were no patients with ADHD, hyperactive/impulsive type. 33.3% of the patients were also on SSRIs. Most patients had either mild or moderate severity on the CGI‐severity scale. 20% of the patients had borderline severity on CGI‐severity scale while there were no patients who had severely or among most extremely ill on the CGI‐ severity. The CGI‐Improvement scale showed that most patients (60%) had minimally improved score while 13.3% of patients had much improved according to the CGI‐Improvement score. 26.7% of patients did not show any improvement. No side effects were noted.

**Conclusion:** A daily dosage of 400 mg sulbutiamine was well tolerated among all the patients and no side effects were noted. Most patients had a minimal improvement and no worsening of symptoms were noted in patients. Larger studies should be conducted in the future to assess potential long‐term effects of Sulbutiamine in patients with Attention‐deficit hyperactivity disorder. In many countries stimulants are not easily available and potential use of sulbutiamine could be an option.

**Keywords:** sulbutiamine, ADHD, stimulants

**References**

1. Bernado Starling‐Sooes et al. Role of the synthetic B1 Vitamin Sulbutiamine on Health. J. NutriMetab.2020 Apr 20:2020:9349063. doi: 10.1155/2020/9349063
2. Bettendorff, L. 10. Thiamine. In present Knowledge in Nutrition, 11th ed.; Marriot, B., Birt, D.F., Stalling, V., Yates, A., Eds.; Basic Nutrition and metabolism; Academic Press: Cambridge, MA, USA, 2020; Volume 1, p.676. ISBN 978‐0‐323‐66162‐1.
3. Bettendorff, L. Update on Thiamine triphosphorylated derivatives and metabolizing enzymatic complexes. Biomolecules 2021, 11, 1645. (Crossref).

## Retail Access to Cannabis and Cannabis Use During the COVID‐19 Pandemic

Brody Montoya, DO, Victoria Teague, DO

Psychiatry Department, Cape Fear Valley Medical Center, Fayetteville, NC

**Introduction:** The accessibility of cannabis plays a crucial role in shaping its use. Since 2014, the rise of retailers has made cannabis more accessible to the public, leading to increases in both cannabis use and consumption of non‐combustible cannabis in relevant areas. Heightened perceived stress has also emerged as a risk factor for cannabis use. Evidence suggests that increased stress levels during the COVID‐19 pandemic (pandemic) correlate with a rise in overall substance use. Additionally, the pandemic saw increased pulmonary health awareness, with some reports of greater consideration for respiratory risks by people who smoke than before the pandemic. As legislatures contemplate cannabis legalization and retail licensing during an escalating, national mental health crisis, they should deliberate policy impacts on usage behaviors. For clinicians, understanding the motivations driving change in cannabis use during the pandemic can better illustrate strategies for both cessation and harm reduction. This repeated cross‐sectional study investigates variations in cannabis use during the pandemic between two states, New York (NY) and Washington (WA), with similar pandemic responses, but differences in their access to retail cannabis to help guide policymakers and clinicians in the evolving landscape of cannabis legalization.

**Methods:** Data was analyzed from the NY and WA Behavioral Risk Factor Surveillance System (BRFSS) from 2019 and 2021 to compare pre‐pandemic and pandemic levels of cannabis use between a decriminalized/legal cannabis state without retail cannabis, NY, to a state with established retail cannabis, WA. Respondents reported on whether they used cannabis in the past 30 days, and whether smoking was their primary method of cannabis use. Data was analyzed using weighted prevalence and 95% CIs estimated using a bootstrap approach, with statistics performed by SPSS statistical software.

**Results:** This study analyzed 75,606 responses, with a median age range of 45‐54 years old, 53.8% of responses from those who identify as female, and the following race/ethnicity profile: 74.2% White, 6.1% Black, 8.9% Hispanic, and 10.8% Other (American Indian or Alaskan Native, Asian, Native Hawaiian or Pacific Islander, Other, Multiracial, and Not Reported). For cannabis use in the past 30 Days, NY and WA had a prevalence of 8.2% (7.8% to 8.7), and 14.8% (14.1% to 15.5%) respectively in 2019, with a change of ‐5.1%, (‐4.6% to ‐5.6%) and ‐0.7%, (.2% to ‐1.7%) in 2021. For smoking as a primary method of use in those with cannabis use in the past 30 days, NY and WA had a prevalence of 73%, (70.3% to 75.4%) and 63.3 % (60.8% to 65.8%) respectively in 2019, with a change of ‐1.9%, (1.8% to ‐5.6%) and ‐4.5% (‐1.2% to ‐8.0%) in 2021.

**Discussion**: Our findings indicate a decrease in overall cannabis use and smoking as a primary method of use in both NY and WA populations, despite increased stress during the pandemic. NY demonstrated a lower initial prevalence of cannabis use, experiencing a more substantial decline during the pandemic. WA reported a lower initial prevalence of smoking as the primary method of cannabis use, with a more pronounced reduction during the pandemic. These variations in prevalence and usage align with existing research highlighting access as a determinant of cannabis use. Additionally, the respective shifts away from combustible cannabis may be from the availability of non‐combustible cannabis in the context of pulmonary health considerations. Surprisingly, neither state exhibited significant increases in cannabis use, contradicting some prior research regarding pandemic substance use. As policymakers contemplate cannabis legalization, they should consider the health implications tied to increased cannabis use post‐legalization and funding pro‐social alternatives amid escalating mental health burdens. Furthermore, if legalized, policymakers must consider the impact of accessibility factors such as zoning allowances and maximum retailer density when legislating retail cannabis. For clinicians, these findings underscore the importance of education regarding cannabis use risks and discussions that integrate harm reduction concepts.

**Keywords:** Cannabis, COVID‐19 Pandemic, Public Health

**References**

Black JC, Amioka E, Iwanicki JL, et al. Evaluation of Cannabis Use Among US Adults During the COVID‐19 Pandemic Within Different Legal Frameworks. JAMA Netw Open. 2022;5(11):2240526.
Goodman S, Wadsworth E, Leos‐Toro C, et al. Prevalence and forms of cannabis use in legal vs. illegal recreational cannabis markets. International Journal of Drug Policy. 2020;76(1):102658.
Miller K, Laha‐Walsh K, Albright D, et al. Cannabis use during the COVID‐19 pandemic: results from a longitudinal study of Cannabis users. Journal of Substance Use. 2022; 27(1): 38‐42.
Schauer GL, Dilley JA, Roehler DR, et al. Cannabis sales increases during COVID‐19: Findings from Alaska, Colorado, Oregon, and Washington. International Journal of Drug Policy. 2021;98:103384
Vanderbruggen N, Matthys F, Van Laere S, et al. Self‐Reported Alcohol, Tobacco, and Cannabis Use during COVID‐19 Lockdown Measures: Results from a Web‐Based Survey. Eur Addict Res. 2020;26(6):309‐315.

## Evaluating Stress and Burnout in Medical School Administrative Staff: A Survey Study in a California Allopathic Medical School

Jonathan Shaw, Charles Lai, Peter Bota, Jonathan Townsend

California University of Science and Medicine

**Introduction:** While Burnout in Healthcare is a major issue currently, there has been limited research on the well‐being of the support and administrative workers that are a crucial part of the medical system. The literature on medical school administrative staff (MSAS) burnout is further complicated by different definitions of “staff,” which can also encompass faculty and medical staff. This study aims to provide some insights into the various stressors MSAS face by examining the prevalence of and correlations between burnout factors in MSAS at a California allopathic medical school.

**Methods:** 120 MSAS employees, defined as employees in non‐curricular roles, were contacted anonymously via email list to participate in this study. Two identical survey rounds were sent on February 8^th^ and March 8^th^ 2023, with each form remaining open for responses for 4 weeks. Each survey was broken into 3 sections containing, respectively, a 13‐question proprietary perception scale, 2 questions from the 2021 AAMC Staff Engagement Survey and 3 proprietary questions about MSAS‐specific aspects of their work, and the 19‐question‐long Copenhagen Burnout Inventory (CBI). Due to privacy and retaliation concerns, no demographic questions were included.

**Results:** The Kolmogorov‐Smirnov test indicated that the CBI Client‐related (p = .083), Work‐related (p = .189), and Personal (p = .082) burnout scores are normally distributed, unlike all of the proprietary data. No significant differences between survey rounds was found in either the One‐Way ANOVA of Personal (p = .654), Work‐related (p = .354), and Client‐related (p = .272) subscales or the Kruskal‐Wallis tests of proprietary question responses. Personal and Work‐related (r(41) = .891, p < .001), Personal and Client‐related (r(41) = .692, p < .001), and Work‐related and Client‐related burnout scores (r(41) = .831, p < .001) were found to correlate by Pearson’s correlation. Multiple of the proprietary questions were found to correlate by Spearman’s correlation. The means for the Personal (57.1) and Work (53.4) indicated burnout (CBI cutoff is score ≥50), whereas the Client subscale mean is much less (42.4), albeit the sample size is too small to be conclusive.

**Conclusions:** In light of limited burnout research on MSAS, this study provides valuable insights on the prevalence of burnout in medical school administration. In particular, the unusually high level of Personal and Work‐related burnout among MSAS implies that the high levels of stress in healthcare may have as much to do with systemic and cultural factors as with the inherent stress of medical work. The consistent correlations and distinctions in subscales indicate that burnout is a lasting issue that need to be solved with plans tailored to each role and situation. Especially given the wide variety of MSAS roles, tailored interventions are crucial in supporting this essential but overlooked portion of the medical school and healthcare workforce.

**Keywords:** Burnout, Medical School Administrative Staff, Mental Health

**References**

1. Manaf, M. R. A., Shaharuddin, M. A.‐A., Nawi, A. M., Tauhid, N. M., Othman, H., Rahman, M. R. A., Yusoff, H. M., Safian, N., Ng, P. Y., Manaf, Z. A., Kadir, N. B. A., Yanasegaran, K., Basir, S. M. A., Ramakrishnappa, S., Ariff, M. I., & Ganasegeran, K. (2021). Perceived Symptoms of Depression, Anxiety and Stress amongst Staff in a Malaysian Public University: A Workers Survey. International Journal of Environmental Research and Public Health, 18(22). https://doi.org/10.3390/ijerph182211874
2. Marijanovic, I., Kraljevic, M., Buhovac, T., Ceric, T., Mekic Abazovic, A., Alidžanovic, J., Gojkovic, Z., & Sokolovic, E. (2021). Use of the Depression, Anxiety and Stress Scale (DASS‐21) Questionnaire to Assess Levels of Depression, Anxiety, and Stress in Healthcare and Administrative Staff in 5 Oncology Institutions in Bosnia and Herzegovina During the 2020 COVID‐19 Pandemic. Medical Science Monitor?: International Medical Journal of Experimental and Clinical Research, 27, e930812. https://doi.org/10.12659/MSM.930812
3. Guidetti, G., Converso, D., Sanseverino, D., & Ghislieri, C. (2022). Return to Work during the COVID‐19 Outbreak: A Study on the Role of Job Demands, Job Resources, and Personal Resources upon the Administrative Staff of Italian Public Universities. International Journal of Environmental Research and Public Health, 19(4). https://doi.org/10.3390/ijerph19041995

## Beyond Dopamine: Antipsychotics, Inflammation, Adherence and COVID‐19

Luiz Dratcu, MD PhD FRCPsych^1^, Xavier Boland, BMBS MRCP MRCPsych^2^

^1^Consultant Psychiatrist, Maudsley Hospital, South London and Maudsley NHS Foundation Trust, Denmark Hill, London SE5 8AZ, United Kingdom

^2^Consultant Psychiatrist, Lewisham Hospital, South London and Maudsley NHS Foundation Trust, Denmark Hill, London SE5 8AZ, United Kingdom

From the outset of the COVID‐19 pandemic, concerns have been raised regarding the vulnerability of patients with severe mental illness (SMI) to complications of SARS‐COV‐2 infection. Initial epidemiological findings indicated a strong link between SMI and poor COVID‐19 outcomes. Studies derived from electronic healthcare databases suggested an association between exposure to antipsychotics and increased morbidity and mortality in COVID‐19 [1], prompting a debate around the risk of antipsychotic treatment during the pandemic.

In contrast to database analysis, reports arising from the clinical setting, along with a few clinical targeted studies, showed that patients with SMI receiving antipsychotic treatment fared surprisingly well following exposure to COVID‐19, both in community and inpatient settings [2][3]. Moreover, post‐publication appraisal of the earlier epidemiological studies also revealed methodological flaws which could have compromised their results[4]. Thus, contrary to initial warnings, could emerging clinical evidence be indicating that antipsychotics are actually protective against COVID‐19? [5]

The pathophysiology of severe COVID‐19 is understood to involve uncontrolled hyperinflammatory responses to the SARS‐CoV‐2 virus, or cytokine storms. In the lungs, the ACE2 receptor, the principal receptor for the SARS‐CoV‐2 virus, is highly expressed on epithelial cells, through which the virus enters the organism. Mass infiltration of immune cells into the alveoli and bronchial mucosa, mediated by pro‐inflammatory cytokines such as IL‐6, IL‐1, IL‐17 and TNFa, causes engorgement of pulmonary vasculature and interstitial tissues and later acute respiratory distress syndrome (ARDS). The systemic spread of hyper‐inflammation and subsequent multiorgan failure are together major causes of death in COVID‐19.

Antipsychotics are thought to act by antagonising dopamine D2 receptors along the brain’s dopamine pathways. Yet there is mounting evidence that antipsychotics are also endowed with anti‐inflammatory properties, such as reducing IL‐6 activity. Evidence that cytokines could be influenced by antipsychotic treatment has lent support to the notion that the anti‐inflammatory action of antipsychotics may contribute to the treatment of schizophrenia itself [6]. Arguably the anti‐inflammatory properties of antipsychotics may also attenuate the normal defensive function of the immune system and thus inhibit uncontrolled inflammatory responses such as seen in severe COVID‐19.

Clinical monitoring of patients with SMI during the pandemic has demonstrated that antipsychotics may offer a dual protective effect against COVID‐19. First, by treating psychotic symptoms, antipsychotics may reduce vulnerability to COVID‐19 associated with SMI. Secondly, through their own direct anti‐inflammatory action, antipsychotics may hinder cytokine storms and protect patients with SMI from COVID‐19 complications. Accordingly, now more than ever, patients with SMI should be fully supported in adhering to antipsychotic treatment.

It has also highlighted that, unless specific criteria are used, scrutiny of data from electronic health records might not always reflect the multiple and complex needs associated with SMI and should therefore be challenged. Illness severity, poor insight, negative attitude towards medication and troublesome side effects all contribute to widespread non‐adherence to treatment in SMI. Unwarranted concerns about safety of antipsychotics in the context of the pandemic could have only exacerbated patients’ reticence to continue treatment, thereby increasing their risk of relapsing and experiencing severe COVID‐19.

**Keywords:** Antipsychotics, Inflammation, COVID‐19

**References**

[1] Vai B, Mazza MG, Delli Colli C, Foiselle M, Allen B, Benedetti F et al. Mental disorders and risk of COVID‐19‐related mortality, hospitalisation, and intensive care unit admission: a systematic review and meta‐analysis. Lancet Psychiatry 2021; 8: 797– 812.
[2] Boland X, Dratcu L. COVID‐19 and acute inpatient psychiatry: the shape of things to come. Int J Psychiatry Clin Pract. 2021 Jun;25(2):132‐134. doi: 10.1080/13651501.2020.1801755. Epub 2020 Aug 5. PMID: 32755472.
[3] Boland X, Dratcu L. Clozapine in the Time of COVID‐19. Clin Psychopharmacol Neurosci. 2020 Aug 31;18(3):450‐453. doi: 10.9758/cpn.2020.18.3.450. PMID: 32702224; PMCID: 202 PMC7383003.
[4] Boland X, Dratcu L. Antipsychotics and COVID‐19: the debate goes on. Lancet Psychiatry. 2021;8(12):1030. doi: 10.1016/S2215-0366(2100396-5)
[5] Dratcu, L., Boland, X. Can antipsychotic use protect from COVID‐19? Schizophrenia Research. 2021, 236, 1‐2.
[6] Mondelli V, Howes O. Inflammation: its role in schizophrenia and the potential antiinflammatory effects of antipsychotics. Psychopharmacology (Berl). 2014 Jan;231(2):317‐8. doi: 10.1007/s00213-013-3383-3. PMID: 24337065.

